# Why Post-Cardiac Arrest Interventions Often Fail: Therapeutic Amenability and the Rapidly Closing Window of Neuroprotection

**DOI:** 10.3390/jcm15124496

**Published:** 2026-06-10

**Authors:** Jae Hoon Lee

**Affiliations:** Department of Emergency Medicine, Dong-A University College of Medicine, Busan 49201, Republic of Korea; leetoloc@dau.ac.kr; Tel.: +82-51-240-5590

**Keywords:** heart arrest, out-of-hospital cardiac arrest, hypoxia-ischemia, brain, targeted temperature management, hypothermia, induced, time-to-treatment, cerebrovascular circulation, severity of illness index, neuroprotection, randomized controlled trials as topic

## Abstract

Hypoxic–ischemic brain injury remains the leading cause of death and neurological disability after cardiac arrest. Although targeted temperature management (TTM) and other neuroprotective strategies have demonstrated promising results in preclinical studies, large randomized controlled trials have largely failed to show consistent clinical benefit. This review examines two major limitations that may contribute to these translational failures: delayed initiation of therapy beyond a time-limited therapeutic window and the lack of baseline injury severity stratification. Evidence from both experimental and clinical studies suggests that the opportunity to modify neurological injury may be confined to the first few hours after return of spontaneous circulation (ROSC). Delayed intervention may occur after irreversible neuronal injury, microvascular dysfunction, and impaired cerebrovascular autoregulation have already become established. In addition, cardiac arrest survivors represent a heterogeneous population. Patients with minimal injury may recover with standard supportive care, whereas those with severe irreversible injury are unlikely to benefit from neuroprotective interventions. Patients with moderate-severity brain injury may represent the subgroup most likely to respond to targeted therapies. Ultra-early stratification using neuroimaging, electroencephalography, circulating biomarkers, and clinical risk scores may help identify patients with therapeutic potential. This review proposes that future post-cardiac arrest research should integrate both time-sensitive intervention strategies and early injury severity stratification. Large prospective studies and randomized controlled trials are needed to determine not only whether interventions are effective, but also when they should be initiated and which patients are most likely to benefit.

## 1. Introduction

Hypoxic–ischemic brain injury is the principal determinant of death and disability after cardiac arrest. Targeted temperature management (TTM) has demonstrated outstanding neuroprotective effects in preclinical animal experiments [1,2], in which the key determinants of its therapeutic effect—timing of treatment (very early), temperature, duration, and the degree of injury (severity)—have been meticulously elucidated [2]. However, large-scale randomized controlled trials have repeatedly failed to reproduce this benefit, and recent guidelines have replaced the concept of TTM with the term temperature control and recommend individualized care targeting the prevention of fever [3,4]. Parallel translational failures have been reported for interventions targeting mean arterial pressure (MAP) and oxygen delivery. This review proposes that two underappreciated factors largely explain these discrepancies: the failure to intervene within a narrow, time-limited therapeutic window, and the absence of baseline injury-severity stratification. These major trials therefore warrant systematic re-examination.

This review is based on a focused examination of the literature identified in PubMed over a three-month search period using combinations of search terms including “cardiac arrest”, “resuscitation”, “temperature management”, “mean arterial pressure”, and “blood gas”. It investigates when therapeutic interventions are required and in which patient subgroups they are effective, with the goal of improving survival and favorable neurological outcomes after cardiac arrest. It further explores a practical clinical approach for selecting patients likely to benefit from intervention on the basis of ultra-early severity assessment.

## 2. Overview of Major Clinical Trials in Post-Cardiac Arrest Care

Following the success of preclinical animal studies, the 2002 clinical trials in patients (the Hypothermia After Cardiac Arrest [HACA] Study Group trial [5] and the Bernard et al. hypothermia study [6]) produced favorable results, and hypothermia therapy appeared to become a standard treatment. However, from the TTM Trial in 2013 through FROST-I Trial in 2018 [7] and the TTM2 Trial in 2021 [8], large-scale randomized controlled trials failed to demonstrate a benefit of TTM (Table 1). The HYPERION Trial in 2019 was the only large-scale randomized controlled trial (RCT) in cardiac arrest patients with non-shockable rhythms to demonstrate a benefit of TTM; however, it has been criticized because its fragility index was extremely low (1), rendering its statistical significance highly unstable, and because the in-hospital cardiac arrest study HYPERION-IHCA Trial was terminated early, raising concerns regarding the robustness of the findings [9,10].

The therapeutic effect of targeted temperature management is grounded in cerebral physiological principles, and based on these physiological mechanisms, further consideration is required not only for temperature control but also for the effects of regulating MAP and oxygen concentration. In patients with traumatic brain injury, a MAP > 80 mmHg is recommended [11], whereas in cardiac arrest patients, a threshold of >65 mmHg is recommended [3]. This discrepancy largely reflects the fact that most RCTs investigating MAP targets in cardiac arrest have failed to demonstrate a benefit of higher MAP levels (Table 2) [12,13,14,15]. However, it has also been argued that, just as MAP > 80 mmHg is necessary in traumatic brain injury due to disrupted autoregulation, MAP targets of 85 to 100 mmHg should be considered in hypoxic–ischemic encephalopathy [16]. That trial also reported improved cerebral oxygenation with higher MAP. In addition, reviews of studies that derived optimal MAP have shown that the mean optimal MAP is generally above 80 mmHg [17,18]. However, similar to TTM studies, research on MAP interventions has not adequately addressed the issues of the time-limited therapeutic window and severity stratification. These same limitations are also present in studies investigating O_2_ and CO_2_ interventions. It is therefore necessary to re-examine the key characteristics of studies on therapeutic interventions in post-cardiac arrest care and to consider both the effective timing of interventions to improve neurological outcomes and the patient subgroups that are most likely to respond to treatment.

**Table 1:** TTM studies.

General Studies (Strong Evidence)						
Study (Year)	Study Type	TTM Intervention	Inclusion Criteria (Disease Severity)	Baseline Severity Stratification (Disease Severity)	Time Interval from Cardiac Arrest to TTM Start	Neurological Outcome *
Le May et al. [19]	RCT	31 vs. 34 °C	GCS ≤ 8	No (shock 33–39%, shockable 86%, lactate 4.4–4.6 mmol/L, pH 7.3)	204–228 min	no significant difference
Dankiewicz et al. [8]	RCT	33 vs. 37.5 °C	FOUR motor < 4 (excluded unwitnessed asystole)	No (no flow time ≤ 1 min, shock 28–30%, shockable 72–75%, lactate 5.8–5.9 mmol/L, pH 7.2)	133–136 min	no significant difference
Lascarrou et al. [20]	RCT	33 vs. 37 °C	GCS ≤ 8 (non-shockable no-flow < 10 min low-flow < 60 min)	No (shock 56–61%, shockable 0%)	219–233 min	difference 4.5% †
Lopez-de-Sa et al. [7]	RCT	32 vs. 33 vs. 34 °C	comatose (witnessed shockable no-flow < 20 min low-flow < 60 min)	No (BP 87–94 mmHg, lactate 5.5–6.4 mmol/L, pH 7.19–7.22)	157–167 min	no significant difference
Debaty et al. [21]	RCT	EMS-cooling vs. hospital-cooling ‡	received advanced life support	No (SAPS II 82–85 §, lactate 7–8.2 mmol/L, pH 7.1)	49–127 min from cardiac arrest to 34 °C	no significant difference
**Stratified Studies (Weak Evidence)**						
Nutma et al. [22]	post hoc prospective cohort	33 vs. 36 °C	GCS ≤ 8	Moderate encephalopathy at arrest 12 and 24 h	(TTM induced as soon as possible)	OR 2.39
Nishikimi et al. [23]	prospective cohort	33–34 vs. 35–36 °C	GCS ≤ 8	6 < rCAST < 14 before TTM	–	OR 1.7
Callaway et al. [24]	retrospective cohort	33 vs. 36 °C	Comatose (excluded cerebral edema and highly malignant EEG)	PCAC 3, 4 within ROSC 6 h	(TTM induced as soon as possible)	RR 1.37, 1.76
Okazaki et al. [25]	post hoc prospective cohort	32–34 vs. 35–36 °C	Comatose	lactate > 12 mmol/L after ROSC ‖	134–226 min	OR 2.31

* Neurological outcomes were defined differently across studies: CAPITAL-CHILL used a Disability Rating Scale (DRS) score > 5; TTM2 used a modified Rankin Scale (mRS) score ≥ 4; HYPERION used a Cerebral Performance Category (CPC) score ≤ 2; FROST-I used an mRS score ≤ 3; Debaty et al. [21]. assessed outcomes using neuron-specific enolase (NSE), survival, and CPC scores; Nutma et al. [22], Nishikimi et al. [23], and Okazaki et al. [25]. used a CPC score ≤ 2; and Callaway et al. [24]. used a CPC score ≤ 3. † Fragility index 1. ‡ Targeted temperature 32–34 °C. The hypothermia group was not compared with the normothermia group. § The Simplified Acute Physiology Score II (SAPS II) is a validated severity-of-illness scoring system. A SAPS II score ≥ 80 is associated with a mortality rate exceeding 70%. Independent of the PCAC score, patients with GWR < 1.2 or a malignant EEG pattern had a 96% mortality rate regardless of the TTM target temperature. ‖ TTM was withdrawn in 161 patients due to severe hypotension, refractory arrhythmia, or an anticipated poor prognosis.

**Table 2:** MAP studies.

Neutral Studies (Strong Evidence)						
Study (Year)	Study Type	MAP Intervention	Inclusion Criteria (Disease Severity)	Baseline Severity Stratification (Disease Severity)	Time Interval from Cardiac Arrest to MAP Intervention	Neurological Outcome *
Kjaergaard et al. [15]	RCT	63 vs. 77 mmHg	comatose (excluded unwitnessed asystole)	No (shockable 84–86%, lactate 5.6–6.1 mmol/L, pH 7.2)	>146 min (after ICU admission)	no significant difference
Grand et al. [12]	RCT	<70 vs. 70–80 vs. >80 mmHg	comatose GCS ≤ 8	No (shockable 76–85%, lactate 5–7 mmol/L)	(after ICU admission)	no significant difference
Ameloot et al. [16]	small RCT	>65 vs. 85–100 mmHg	GCS < 8	No (shockable 65–69%, lactate 6.2–6.6 mmol/L)	(after ICU admission)	no significant difference
Jakkula et al. [14]	small RCT	65–75 vs. 80–100 mmHg	comatose GCS motor < 5	No	>191–193 min (after ICU admission)	no significant difference
**Positive Studies (Weak Evidence)**						
Roberts et al. [26]	prospective cohort	70–90 vs. >90 mmHg	comatose GCS motor < 6	No (shockable 37%)	>15 min	RR 2.46

* Neurological outcomes were defined differently across studies: BOX used a CPC score ≥ 3; ENDO-RCA used NSE and an mRS score ≥ 4; NEUROPROTECT used a CPC score ≤ 2; COMACARE used NSE and a CPC score ≥ 3; and Roberts et al. [26]. used an mRS score ≤ 3.

## 3. Mechanisms of Brain Injury After Cardiac Arrest and Neuroprotective Mechanisms of Interventions

Ischemic brain injury after cardiac arrest occurs through two principal mechanisms. Primary neuronal injury develops during the no-flow and low-flow periods, when interruption of cerebral blood flow (CBF) leads to ischemic depolarization and failure of ion pumps in the neuronal cell membrane. As a result, neurons begin to die through mechanisms such as cytotoxic edema and excitotoxicity [9,27]. This is followed by secondary injury after return of spontaneous circulation (ROSC), during which reperfusion, repolarization, and subsequent dysregulation cause further damage [10]. Ongoing neuronal injury is driven by mechanisms including microvascular dysfunction (i.e., vasoconstriction, blood–brain barrier disruption, and increased viscosity due to microthrombi), increased cerebral metabolism, disrupted autoregulation, elevated intracranial pressure, and hypoxemia [27,28].

TTM is known to exert neuroprotective effects by acting on these secondary injury mechanisms. For every 1 °C reduction in body temperature, cerebral metabolism decreases by 5 to 10%, thereby protecting neurons; hypothermia also prevents mitochondrial dysfunction, reduces apoptosis, and decreases inflammatory mediators, thereby attenuating excitotoxicity [27]. Hypothermia is also reported to improve neurological outcomes by preventing microvascular constriction [29]. In the injured brain, restoration of flow at the level of microvessels and capillaries—capillary recirculation rather than large-vessel reperfusion—is crucial for neuronal recovery [30]. Hypothermia also preserves blood–brain barrier (BBB) integrity [31], and reduces cerebral edema and CBF, thereby significantly lowering intracranial pressure (ICP) [32]. Compared with normothermia, hypothermia transiently increases CBF (hyperemic phase) followed by a decrease (hypoperfusion phase) [30]. Even when CBF is further reduced under hypothermia, autoregulation is better preserved and microvascular dysfunction is less pronounced. Hypothermia widens the autoregulation window and maintains vasoreactivity even at lower CBF levels [33].

Maintaining an appropriate MAP and oxygen delivery is a key therapeutic strategy to secure adequate cerebral perfusion pressure (CPP) in the setting of impaired cerebrovascular autoregulation and thereby prevent secondary cerebral tissue hypoxia. CPP is defined as MAP minus ICP (CPP = MAP − ICP) [34]. Under normal conditions, the brain maintains CPP through autoregulation by adjusting vasoreactivity and ICP across a MAP range of approximately 50 to 150 mmHg. However, approximately 44% of cardiac arrest patients exhibit impaired autoregulation, and about 28% demonstrate a right-shifted and narrowed autoregulation window [35]. Only about 28% of patients retain normal autoregulation. NIRS-based studies of cerebrovascular reactivity have also shown that autoregulation is impaired in approximately 70% of cardiac arrest patients [36]. When the autoregulation window becomes narrowed, right-shifted, or lost, CPP may be reduced even at normal blood pressure, thereby aggravating brain injury. Indeed, in a rat model of cardiac arrest, CBF decreased markedly despite the maintenance of normal MAP [37]. Higher CBF does not necessarily induce neurological recovery from brain injury. Microvascular dysfunction can lead to vasoparalysis and persistent cerebral hyperemia, which is associated with poor neurological outcomes [38,39]. Delayed hyperperfusion in which CBF exceeds the cerebral metabolic rate of oxygen (CMRO_2_) is referred to as luxury perfusion. In animal models of cardiac arrest that demonstrated neurological improvement, reactive hyperemia was observed, characterized by an initial increase in CBF during the first 20 min followed by a subsequent decline. In rat experiments, after 15 min of ischemia, most recovery occurred following 60 to 90 min of reperfusion [40].

The benefit of inducing higher MAP beyond normal reperfusion remains uncertain in animal studies. Although higher MAP was favorable for improving cerebral perfusion and preserving autoregulation, no clear differences in ischemic markers were observed [41]. In a resuscitated porcine model of cardiac arrest, a higher MAP improved CPP but aggravated neuroinflammation and led to delayed impairment of autoregulation [42]. The period during which MAP intervention may be required corresponds to the no-reflow and delayed hypoperfusion phases, when CBF is lower than CMRO_2_. Increased vascular resistance caused by swelling of perivascular cells, capillary collapse, microthrombi, and increased blood viscosity may necessitate transiently higher MAP to overcome microvascular resistance [9,43]. A key consideration is that when ICP is elevated, a higher MAP may help maintain CPP; however, in the setting of impaired autoregulation, a higher MAP—while still maintaining CPP—may generate supra-CPP flow and thereby exacerbate vasogenic edema [44]. Because good neurological outcome is possible even when early autoregulation is impaired [45], identifying an optimal MAP is crucial. Rather than simply inducing a higher MAP, efforts should be made to identify an optimal MAP according to the status of ICP and autoregulation. On the other hand, an adequate MAP can help maintain oxygen delivery in accordance with increased cerebral metabolic demands [44]. Even after ROSC, secondary cerebral tissue hypoxia frequently occurs due to microcirculatory dysfunction, impaired autoregulation, and mitochondrial dysfunction; therefore, adequate oxygen supply is essential for the recovery of ATP synthesis in injured neurons [9,10]. Conversely, excessive oxygen delivery increases reactive oxygen species production and may aggravate secondary brain injury through mitochondrial dysfunction, cerebral vasoconstriction, and DNA damage. In animal experiments, ventilation with 100% oxygen resulted in more severe neurological deficits and neuronal injury [10].

## 4. Hemodynamic Monitoring and Support in Cardiac Arrest Patients

Another cornerstone of post-cardiac arrest care is fundamental hemodynamic monitoring and support. Much like septic or cardiogenic shock, shock in PCAS is a multifactorial phenomenon, involving a mixture of hypovolemic, cardiogenic, and distributive components that can evolve over time. Utilizing echocardiography to identify the primary shock subtype and implementing targeted therapy is crucial. In cardiac arrest patients, it is well-known that early ejection fraction (EF) can recover even if initially low [46]. This is a transient phenomenon caused by cardiac stunning, which occurs in two-thirds of ROSC patients and typically resolves within 3 days [47]. In patients with distributive shock and a relatively preserved EF, fluid therapy is associated with improved neurological outcomes; however, in cases of cardiogenic shock with an EF of 40% or less, fluid volume shows no correlation with prognosis [48]. Therefore, echocardiography should be used to distinguish patients who require aggressive fluid resuscitation from those who do not. Relying solely on EF to guide fluid and vasopressor therapy can be misleading; clinical decisions should be based on an integrated interpretation of the available data. Since an increase in cardiac filling pressure (i.e., increased E/e’) may be more closely associated with poor prognosis than a decrease in EF [46,47], it is advisable to assess E/e’ alongside EF. Clinicians should refer to various clinical data when deciding whether to restrict fluids and initiate vasopressors. While fluid responsiveness based on dynamic parameters provides a more accurate guide for fluid therapy, its practical application remains challenging [49]. Research suggests that while fluid resuscitation is necessary during the initial arrest phase, subsequent fluid restriction is beneficial for survival. In one study, the 7-day cumulative I/O was +3600 mL in the mortality group compared to −140 mL in the survivor group, with a hazard ratio of 2.2 for the group with higher cumulative fluid balance [50]. Similar beneficial effects of post-resuscitation fluid restriction have been observed in patients with septic shock [51]. Once subtype-specific hemodynamic support is initiated via echocardiography, continuous monitoring is essential. Diastolic blood pressure (DBP) has been found to reflect the prognosis of cardiac arrest patients more accurately than systolic blood pressure (SBP) or MAP [52,53]. Furthermore, a ΔPCO_2_ > 6 mmHg, increased lactate within the first 12 h [54], and a Δlactate < 10%/2 h [54] are excellent indicators of inadequate perfusion. These parameters should be interpreted integratively alongside capillary refill time and ScvO_2_ [54]. Utilizing these indicators to monitor cardiac arrest patients can help prevent fluid overload and the excessive use of vasopressors. In conclusion, hemodynamic support should be tailored during and after acute resuscitation, guided by integrative echocardiographic assessments (including EF and E/e’) and diverse clinical data. It is crucial to monitor DBP, ΔPCO_2_, and lactate kinetics, and to actively implement de-resuscitation (fluid removal) in critically ill patients with evidence of fluid accumulation [55].

## 5. Failure of Large RCTs and the Time-Limited Therapeutic Window

Why has the neuroprotective effect of TTM interventions observed in animal studies failed to be replicated in human clinical trials? The primary reason, as noted in numerous review studies, is the failure to reach the target temperature within 4 to 6 h of ROSC. In large-scale RCTs, the time from ROSC to TTM initiation ranged from 113 to 233 min (Table 1), yet the time to achieve the target temperature exceeded 5 h [10]. This stands in stark contrast to animal experiments, where target temperatures are often reached within approximately 30 min post-ROSC. In a rat model of 10 min asphyxial cardiac arrest, therapeutic hypothermia at 33 °C initiated within 4 h of ROSC maintained a 19% rate of good neurological outcomes. However, when TTM was delayed until 8 h post-ROSC, hippocampal neuronal survival was 65% and the 7-day survival rate was 14%, but the rate of good neurological outcomes dropped to 0% [56]. Although TTM exerts neuroprotective effects even beyond 4 h post-ROSC, achieving functional neurological recovery may be most effective when initiated intervention within a narrow window of less than 4 h. Similarly, studies in canine models have reported that a cooling delay of just 15 min significantly exacerbates neurological deficits [57]. A meta-analysis of 181 animal studies further suggested that the maximum therapeutic window for TTM is within 6 h [58]. Regarding human studies, a meta-analysis of RCTs on TTM conducted between 2001 and 2022 reported that initiating TTM within 2 h of the event and maintaining it for at least 24 h significantly improved neurological outcomes in patients with shockable rhythms. In contrast, studies in which TTM was initiated more than 2 h after the event demonstrated no significant improvement in prognosis [59]. Furthermore, a meta-analysis of human studies emphasized that the time to reach target temperature is more critical than the time of TTM initiation [60]. Even the study that achieved the most rapid time to target temperature among those included in the meta-analysis—reaching 34 °C within 49 to 127 min from the onset of cardiac arrest—failed to demonstrate a significant improvement in clinical outcomes [21]. However, this study did not include a normothermic control group and enrolled a severely ill population (SAPS II scores of 82 to 85) with a predicted mortality exceeding 70%, which may have masked any meaningful differences. In patients undergoing extracorporeal cardiopulmonary resuscitation (ECPR), the benefits of TTM may be more pronounced due to the ability to initiate intervention and achieve target temperatures rapidly. A meta-analysis of retrospective studies focusing on ECPR patients found that in highly severe cases, lower temperatures (moderate vs. mild hypothermia and moderate hypothermia vs. normothermia) were associated with improved neurological outcomes [61]. However, a network meta-analysis showed no significant effect, likely due to high heterogeneity and a paucity of studies comparing all three groups (moderate hypothermia, mild hypothermia, and normothermia). Given the retrospective nature of these observations, these findings only suggest the potential benefits of TTM.

Similarly, RCTs on MAP interventions likely missed the critical therapeutic window, as interventions typically commenced only after ICU admission (Table 2) [44]. Early intervention is crucial because irreversible neurological damage can manifest as early as 2.7 h post-ROSC [62], and luxury perfusion may occur by 4 h post-ROSC [63]. These findings suggest that without ultra-early intervention, microvascular obstruction may occur, rendering neurological damage inevitable. To understand the role of MAP, it is essential to comprehend the changes in CBF following resuscitation. Following cardiac arrest, the brain undergoes ischemic depolarization followed by reperfusion repolarization, which subsequently evolves into a dysregulation phase. During the transition from reperfusion repolarization to dysregulation, a transient hyperemia occurs; in this stage, hyperemic regions coexist with no-reflow areas, the latter of which expand as the duration of ischemia increases [10]. Following the hypoperfusion phase, both cerebral blood flow (CBF) and the cerebral metabolic rate of oxygen (CMRO_2_) gradually return to baseline as the brain transitions from the dysregulation phase to the recovery and repair phase [30,64,65]. In severe neuronal injury, however, impaired autoregulation or microvascular dysfunction may precipitate luxury perfusion or delayed hypoperfusion, producing heterogeneous CBF patterns that may be hyperemic, suppressed, or pseudo-normalized [66,67,68]. Although prospective rather than randomized, a study showed that increasing MAP to approximately 90 mmHg within 15 min of ROSC favored good neurological outcomes (RR 2.6) compared to maintaining MAP at 70 to 90 mmHg [26]. Observational studies in cardiac arrest patients suggest the therapeutic window for MAP intervention is within 6 h of ROSC [26,69,70]. While definitive evidence that early MAP intervention improves neurological prognosis is still lacking, there remains a potential benefit for targeting higher MAP levels immediately after ROSC than currently recommended by guidelines. Ideally, the best approach would be to tailor MAP to the individual patient’s requirements.

Interventions targeting oxygen and carbon dioxide levels have also failed to demonstrate therapeutic efficacy in RCTs (Table 3). In the BOX and TAME trials, interventions were initiated approximately 6.5 h after cardiac arrest, following ICU admission [71,72]. The EXACT study, which maintained higher oxygen concentrations starting 50 min after cardiac arrest, reported increased survival rates but failed to improve neurological outcomes (Table 3). In that study, the EMS ventilators were limited to only two settings—100% oxygen or an air-mix (approximately 60% FiO_2_)—which precluded incremental titration. Furthermore, the SpO_2_ targets of 90% to 94% may have been suboptimally low [73]. Another critical limitation was the significantly higher frequency of hypoxic episodes SpO_2_ < 90%) observed in the 90–94% group prior to ICU admission, which may have confounded the neurological assessments [10].

## 6. Result Differences According to Baseline Injury Severity and Positive Studies

In a porcine cardiac arrest model, assuming a 7 min CPR duration, the probability of ROSC was 75% with a no-flow time of 7 min, 75% to 88% at 7 to 10 min, and 0% at 13 min [74]. In another swine model with 20 min of CPR, the ROSC rate was 83% at a no-flow time of 13 min, but decreased to 50 to 66% at 15 to 17 min [75]. No animals with a no-flow time ≥ 14 min achieved favorable neurological recovery [76]. A notable exception in a canine model demonstrated that inducing a tympanic temperature of 10 °C achieved good neurological outcomes even after a 90 min no-flow interval [77]. Human studies show similar patterns. When the no-flow time exceeded 12 min, the probability of a good neurological outcome was <1% [78]. Furthermore, no patients achieved favorable neurological recovery with a no-flow time of 20 min or longer [79]. Conversely, the threshold for low-flow time remains ill-defined. Among patients achieving Cerebral Performance Category (CPC) 1 or 2, the longest recorded low-flow time was 67 min [80]. Another study indicated that the probability of favorable neurological recovery decreases to less than 1% when low-flow time exceeds 43 min [81]. Nevertheless, in specific populations—such as those with shockable rhythms, witnessed arrests, bystander CPR, age ≤ 60 years, or those receiving ECPR—good neurological outcomes have been observed even with low-flow durations of 67 to 98 min [80,82,83]. While outcomes vary based on patient status and aggressive management, the fundamental relationship remains: prolonged no-flow and low-flow times are strongly associated with refractoriness to therapeutic interventions. Even if ROSC is achieved, patients who have already developed severe encephalopathy due to prolonged ischemia may become refractory to any interventional therapy. Consequently, typical clinical trials, such as the FROST-I study, should ideally include only patients with no-flow times < 20 min and low-flow times < 60 min. The inclusion of patients with refractory, severe encephalopathy makes it increasingly difficult to demonstrate the efficacy of therapeutic interventions. Among the four major RCTs on TTM, the CAPITAL-CHILL study lacked specific criteria for no-flow and low-flow durations [19], while the HYPERION study applied strict criteria (no-flow time < 10 min) [20]. The TTM2 study eliminated the possibility of prolonged no-flow times by excluding patients with unwitnessed asystole [8].

**Figure 1:**
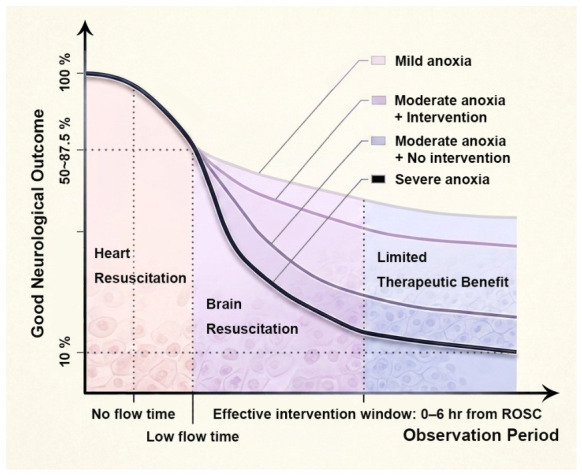
Conceptual graph: Time limited intervention period and severity.

When ROSC is achieved through high-quality CPR, additional baseline injury severity stratification becomes essential. It is crucial to distinguish patients who may benefit from TTM and other interventions from those predicted to have a dismal prognosis, for whom supportive care should be the focus. Several attempts have been made to distinguish such patients through severity assessment. Both mild and severe severity groups may be relatively unresponsive to therapeutic interventions [8,21]. Several studies suggest that a moderate severity group is most likely to benefit from TTM. Potential candidates for TTM efficacy include patients with moderate encephalopathy on EEG monitoring [22], a revised post-Cardiac Arrest Syndrome for Therapeutic hypothermia (rCAST) score between 6 and 14 [23], a Pittsburgh Cardiac Arrest Cardiovascular (PCAC) score of 3 to 4 [24], or lactate levels > 12 mmol/L [25]. While patients at the extremes of severity may derive limited benefit, timely and appropriate intervention in the moderate group could shift their trajectory toward mild severity; otherwise, they may deteriorate toward severe injury (Figure 1). As demonstrated by Nishikimi et al. using rCAST to identify a subgroup in which TTM was meaningful, several retrospective studies have reported similar therapeutic benefits of TTM in moderate severity groups classified by rCAST [84,85]. An RCT using rCAST stratification is currently underway, and their results are highly anticipated [86]. However, when stratified using mCAHP (modified Cardiac Arrest Hospital Prognosis), TTM effects were observed in the mild or severe groups rather than the moderate group [87].

In studies concerning MAP and O_2_ interventions, baseline severity stratification in post-cardiac arrest patients has not been adequately addressed. The prospective study by Roberts et al. is unique among prospective studies and RCTs in demonstrating that a MAP > 90 mmHg resulted in better neurological outcomes compared to a MAP of 70 to 90 mmHg. This study’s strength lies not only in the early initiation of MAP intervention (within 15 min) but also in its selection of a high-severity patient population. Notably, the proportion of shockable rhythms in the Roberts et al. study was 37%, significantly lower than the 65% to 86% reported in other MAP-related RCTs [26]. Most of post-cardiac arrest patients exhibit impaired cerebral autoregulation and rightward shifting of the autoregulation, suggesting the need for higher MAP targets. Elevated intracranial pressure may necessitate even higher MAP levels to maintain cerebral perfusion [88]. However, in the presence of concurrent cardiopulmonary dysfunction or impaired cerebral autoregulation, the potential harms of targeting MAP levels above the patient’s optimal MAP should also be carefully considered. For patients with moderate brain injury, early and targeted optimization of MAP may be particularly important, a hypothesis that warrants confirmation in future studies.

Similarly, RCTs investigating oxygen and carbon dioxide targets did not stratify patients by baseline severity, although the proportion of shockable rhythm ranged from 60% to 85%. The EXACT trial only included patients who maintained an SpO_2_ of 95% during oxygen administration [73], the BOX trial included only patients with a cardiac origin of arrest [71], and the TAME trial excluded unwitnessed asystole [72]. As in ischemic stroke or myocardial infarction, where the concept of the penumbra guides interventional therapy, it is critical to use severity assessment to identify patients with a therapeutic penumbra who remain salvageable through timely intervention.

## 7. Ultra-Early Stratification According to Baseline Injury Severity and Therapeutic Options

For patients with post-cardiac arrest syndrome (PCAS), it is imperative to establish the intensity of therapeutic interventions through ultra-early stratification according to baseline injury severity. More severe brain injury necessitates more aggressive management, such as lower target temperatures, extended treatment durations [2], and higher MAP targets [17]. To achieve this, an accurate assessment of the initial severity of brain injury is required, specifically to identify the moderate-severity group through baseline stratification. Practical tools for this purpose include ultra-early brain MRI, EEG, biomarkers such as neurofilament light chain, and clinical risk scores like rCAST and PCAC. First, Studies have reported that ultra-early brain MRI performed at a mean of 2.7 h post-ROSC demonstrates remarkable prognostic accuracy for neurological outcomes, with an AUC of 0.9 to 0.93 [62]. Specifically, initial detection of injury involving more than 2.4% of the whole brain in cardiac-origin arrests, or more than 13.5% in respiratory-origin arrests, suggests a poor neurological prognosis. These findings provide a crucial clue for identifying brain injury of moderate severity in the ultra-early phase post-ROSC. Although ultra-early brain MRI presents logistical challenges—such as its prolonged acquisition time, high cost, and the need for a dedicated fast-track hospital setting—it could serve as an excellent ultra-early screening tool if further research establishes the clinical significance of classifying brain injury severity in this early window. Second, early EEG has already been utilized in research, such as the study by Nutma et al., to differentiate severity and demonstrate clinical efficacy using 21-channel recordings. Since that study categorized severity based on EEG patterns at 12 and 24 h post-arrest, further randomized controlled trials (RCTs) are needed to evaluate the application of therapeutic interventions based on ultra-early EEG assessments. While multi-channel EEG entails significant setup time, effort, and cost, the global ischemic nature of the brain due to cardiac arrest suggests that analysis of frontal lobe leads alone may provide sufficient diagnostic yield [89]. In particular, a mean suppression ratio between 10% and 36.6% might provide a rationale for identifying the moderate-severity group and warrants further clinical validation [90,91]. However, because such EEG data are influenced by sedatives and patient movement, recordings should be obtained before the administration of sedatives, and careful attention must be paid to the interpretation of muscle artifacts when used for ultra-early severity stratification. Third, biomarkers play a crucial role in assessment. Neuron-specific enolase indicates direct neuronal injury, while glial fibrillary acidic protein reflects astrocyte and BBB damage. Neurofilament light chain can be used to infer axonal injury and white matter degeneration. These biomarkers allow for a more precise estimation of injury severity [92]. For more definitive and simplified criteria, neurofilament light chain levels—which have shown superior prognostic performance compared to S100 and NSE—may potentially be used to categorize the degree of injury [93]. Finally, risk scores offer a simplified assessment method. To date, observational studies have shown that only rCAST and PCAC have successfully differentiated severity with clinically meaningful results. Ultimately, the clinical utility of ultra-early screening tools for stratifying baseline injury severity should be validated through further large-scale prospective studies. Despite recent advances, simple, low-burden, and easily deployable early screening methods for identifying patients according to the severity of neurological injury remain limited.

## 8. Knowledge Gaps and Future Directions

Current research in PCAS care often overlooks the critical integration of a time-limited therapeutic window and baseline injury severity stratification, typically addressing only one or neither of these concepts. There is a pressing need for integrated studies that synthesize these two frameworks. Similar to the management of acute stroke or myocardial infarction, time-sensitive management must be established in PCAS care. This necessitates the implementation of specialized prehospital and hospital system settings to facilitate rapid intervention, though such rigorous protocols may increase the risk of clinical burnout among medical personnel. Identifying the moderate-severity group likely requires a multifaceted approach that integrates the ultra-early tools discussed above—brain MRI, EEG, biomarkers, and risk scores—for comprehensive decision-making, and additional scores such as KORHN, MIRACLE, or TTM may further help identify the moderate-risk group [94]. Despite the logistical challenges of establishing these settings, they are essential prerequisites for advancing care. Improving the prognosis of cardiac arrest survivors depends on prompt interventions facilitated by ultra-early stratification of baseline injury severity. The potential to maximize neurological recovery will only be realized when we can precisely identify the optimal timing and target population for interventions—such as TTM, MAP optimization, and the management of O_2_, CO_2_, fluids, and vasopressors—and apply these baseline severity stratification tools and therapeutic strategies in a highly integrated manner.

## 9. Limitations

This review has several limitations that should be considered when interpreting its conclusions. First, this is a narrative rather than a systematic review; the included literature was not identified through a predefined systematic search strategy, and the selection and interpretation of evidence may therefore be subject to author bias. Second, the evidence supporting a time-limited therapeutic window derives from heterogeneous sources that differ across interventions. The proposed window for TTM—within approximately 4 to 6 h after ROSC—is based largely on experimental animal models, whereas the window for MAP intervention is supported mainly by human observational studies. Animal-derived findings should be extrapolated to patients with caution, given the differences between animal models and the clinical setting, including lower rates of witnessed arrest and bystander CPR, challenges in delivering ultra-early interventions, and the limited capacity for detailed physiological monitoring in humans. The human observational evidence, in turn, is constrained by its non-randomized design, which limits causal inference. Third, the clinical studies cited are heterogeneous in design, patient population, and outcome definition, and several key observations are drawn from retrospective or non-randomized data, further limiting the strength of any causal inference. Finally, the integrated framework proposed here—combining time-sensitive intervention with ultra-early baseline injury-severity stratification—remains hypothesis-generating. The stratification tools discussed, including brain MRI, EEG, circulating biomarkers, and clinical risk scores, have not yet been prospectively validated for guiding therapy in the ultra-early phase. Accordingly, the strategies proposed in this review should be regarded as directions for future investigation rather than established recommendations, and require validation in large-scale prospective studies and randomized controlled trials.

## 10. Conclusions

The therapeutic window for neurological recovery following cardiac arrest may be considerably narrower than previously appreciated. Patients with irreversible severe brain injury are unlikely to benefit from neuroprotective interventions, whereas those with minimal injury may require little beyond standard supportive care. This review highlights the potential importance of initiating interventions—including timely achievement of target temperature, optimization of MAP, regulation of oxygen and carbon dioxide levels, and individualized hemodynamic support—as early as possible, ideally within 4–6 h after ROSC, particularly in patients with moderate-severity brain injury.

However, whether these time-sensitive interventions, guided by early stratification of baseline injury severity, can consistently improve neurological outcomes remains uncertain. The therapeutic window and identification of appropriate patient populations represent recognized limitations of previous post-cardiac arrest trials and remain important challenges for future research. Large-scale prospective studies and randomized controlled trials are needed to validate ultra-early stratification strategies, determine the optimal timing and intensity of interventions, and establish robust clinical evidence for personalized post-cardiac arrest care.

## Figures and Tables

**Table 3:** Blood gas studies.

O_2_ Studies (Strong Evidence)						
Study (Year)	Study Type	Intervention	Inclusion Criteria (Disease Severity)	Baseline Severity Stratification (Disease Severity)	Time Interval from Cardiac Arrest to Intervention	Neurological Outcome *
Bernard et al. [73]	RCT	SpO_2_ 90–94 vs. 98–100%	comatose cardiac origin (at least 95% while > O_2_ 10 L/min or FIO_2_ of 100%)	No (shockable 60–64%, pH 7.17)	52–54 min	no significant difference (survival rate 38.3 vs. 47.9%, *p* = 0.05)
Schmidt et al. [71]	RCT	PaO_2_ 67–75 vs. 97–105 mmHg	comatose (cardiac origin)	No (shockable 85%, lactate 5.8–5.9 mmol/L, pH 7.21)	4.5–6.5 h (after ICU admission)	no significant difference
**CO_2_ Study (Strong Evidence)**						
Eastwood et al. [72]	RCT	CO_2_ 35–45 vs. 50–55 mmHg	comatose (excluded unwitnessed asystole)	No (shock 25.6–29.8%, shockable 70–72.5%, lactate 6.8–7 mmol/L)	6.5 h (after ICU admission)	no significant difference

* Neurological outcomes were defined differently across studies: EXACT used survival and a CPC score ≤ 2; BOX used a CPC score ≥ 3; and TAME used a Glasgow Outcome Scale–Extended (GOS-E) score ≥ 5.

## Data Availability

Not applicable.

## Abbreviations

TTM: targeted temperature management; RCT: randomized controlled trial; MAP: mean arterial pressure; CBF: cerebral blood flow; ROSC: return of spontaneous circulation; BBB: blood–brain barrier; ICP: intracranial pressure; CPP: cerebral perfusion pressure; CMRO2: cerebral metabolic rate of oxygen; ECPR: extracorporeal cardiopulmonary resuscitation; rCAST: revised post-cardiac arrest syndrome for therapeutic hypothermia; PCAC: Pittsburgh cardiac arrest cardiovascular; mCAHP: modified cardiac arrest hospital prognosis; PCAS: post-cardiac arrest syndrome; DBP: diastolic blood pressure; SBP: systolic blood pressure.
